# Conceptual mappings and neural reuse

**DOI:** 10.3389/fnhum.2014.00261

**Published:** 2014-04-29

**Authors:** Cristóbal Pagán Cánovas, Javier Valenzuela Manzanares

**Affiliations:** ^1^Institute for Culture and Society, University of NavarraPamplona, Spain; ^2^English Department, University of MurciaMurcia, Spain

**Keywords:** conceptual mappings, conceptual blending, neural theory of language, neural reuse, metaphor, SNARC effect, material anchors for conceptual blends, mental timeline

## Too much neural reuse, even for a metaphorical brain

Conceptual mappings are correspondances between *conceptual domains* (space, time, force, emotion, etc.) or between entities within the same conceptual domain. Through mapping, we project inferences, elements, and relations from one mental configuration to another. Sets of mappings can become entrenched, creating powerful cognitive habits. For example, across many cultures around the world, temporal relations are conceived by means of spatial relations, in language (“Saturday is almost here”), artifacts (timelines, calendars, sundials), or gesture (Núñez and Sweetser, 2006). Some of the mappings for this template are: duration is spatial extent, events are landmarks, or time is motion along a path.

From over 30 years of conceptual mappings research emerges a picture of the conceptual system as a set of mapping habits. Instead of a static repository of concepts, we have a dynamic network connecting mental structures. Mapping is not exceptional: it is the norm. It is through mapping that concepts are formed, learned, and developed creatively. These ideas have boosted the interest in the most remarkable manifestations of mapping in language and thought: metaphor, metonymy, analogy, counterfactuals, etc.

Metaphor has received far more attention than all the other phenomena combined. Researchers in Conceptual Metaphor Theory (CMT) (Lakoff and Johnson, 1980; Lakoff, 1993), have identified many sets of cross-domain mappings underlying conventional metaphorical expressions: time is space, life is a journey, anger is heat, events are actions, etc. According to CMT, conceptual metaphors are static, ontological, fixed sets of partial correspondances between two conceptual domains. Metaphor transfers inferences from the source domain, more concrete or better structured, to the target domain, which is more abstract or less delineated. A system of thousands of metaphorical mappings constitutes the main mechanism for abstract thought in the human mind.

From the nineties, the semantic postulates of CMT have been used to develop the Neural Theory of Language (NTL) (Gallese and Lakoff, 2005; Feldman, 2008; Lakoff, 2008). In NTL, conceptual metaphors are replaced by neural mappings, combinations of simple neural circuits that carry out conceptual mappings. The major function of all this neural binding is the reuse of sensorimotor brain mechanisms for new roles in language and reasoning. This is congenial with the *grounding* of abstract concepts in perceptual experience (Barsalou, 1999, 2008), called *embodiment* in conceptual mappings research (Johnson, 1987, 2008; Lakoff, 1987; Lakoff and Johnson, 1999). The overarching idea of CMT-NTL is a “metaphorical mind-brain,” based on direct, binary transfer across domains. The transfer is carried out through static cognitive habits, which are implemented by neural circuitry connecting pairs of brain areas. For the brain, metaphor is once more privileged over all other manifestations of mapping, just like it was for the mind. A good example of the rapidly growing interest in the neuroscience of metaphor is the *Frontiers* issue about the topic, currently being edited by Vicky T. Lai and Seana Coulson.

But the metaphorical brain seems quite insufficient to account for the pervasiveness and complexity of neural reuse. In a recent *BBS* target article, Michael Anderson (2010) shows that what is going on in the brain dwarfs the predictions of embodiment or CMT-NTL. Statistics run on thousands of fMRI studies indicate that even fairly small brain regions are typically reused in multiple tasks, with even higher reuse probabilities if a region is involved in perception or action (Anderson et al., 2010).

Neural reuse is ubiquitous and dynamic, and many of its results cannot be explained as domain-structuring inheritance. Anderson examines, among others, the following examples: the SNARC effect (a mental number line with magnitudes increasing from left to right), the correlation between finger representation and numerical cognition, the interaction of word and gesture, or the phonological loop in working memory. These cases present no metaphorical projection, and some of them involve more than two components. Rather than direct transfer of information, a given system seems to be reused for a non-primary purpose because it happens to have a function or structure that are appropriate for the particular cognitive task at hand. As Anderson claims, we need a broader theoretical framework, able to account for those individual phenomena as well as for the general prevalence of neural reuse.

## From transfer to emergence: the network model of conceptual integration

Can conceptual mappings research provide such a framework? For one thing, the CMT-NTL model is certainly not the only one in the field. CMT and the pervasiveness of mapping were the point of departure of Gilles Fauconnier's Mental Space Theory (Fauconnier, 1985, 1997), later developed by Fauconnier and Mark Turner into Conceptual Integration Theory, or Blending Theory (BT) (Turner, 1996, 2014; Fauconnier and Turner, 2002).

Beyond the common ground with CMT (Fauconnier and Lakoff, 2013), BT introduces significant innovations. Mental spaces are not vast domains such as time or space, but small conceptual packets that flexibly combine entrenched structures and contextual information, for local purposes of thought and action. Mappings are established through structural or functional correspondances between input spaces in a generic mental space. The participation of more than two inputs is quite typical. Selectively projected to a blended space or *conceptual blend*, elements from the inputs interact, typically producing emergent structure, which cannot be accounted for by direct transfer between domains. Inferences can take place in the blend, but also be projected back to the inputs, which can be modified as the process unfolds. The mappings, the emergence of novel structure, the adjustment of the inputs, and everything else going on is guided by universal governing principles and competing optimality principles, by the functional requirements of the particular network of mental spaces, dictated by context and goals, and by the creativity of the individual or group who are striving to make the most of it all.

The overarching picture that results from BT bears important differences with that of CMT-NTL. Advanced blending underlies all manifestations of mapping, including metaphor, which is just one more surface product of this species-defining capacity for integrating disparate mental components into new, meaningful wholes. The human brain is a bubble chamber of mental spaces, constantly building new integration networks, and a culture is an even larger bubble chamber (Fauconnier and Turner, 2002, p. 321–322). Just like evolution—and neural reuse—, blending is opportunistic: it reuses whatever is functionally suitable, right there and then. As it happens with the natural selection of living organisms, the process of trial and error in conceptual and neural reuse never stops. Through it, minds and cultures select the few integrations that are really useful, anchor them by means of symbolic procedures, and pass them on to the next generation. To become productive habits, both generic templates of conceptual integration and patterns of neural reuse need to find an adequate niche within the general system (about the notion of *neural niche*, see: Anderson, 2010, and the commentary by Atsushi Iriki therein; Iriki and Sakura, 2008; Iriki and Taoka, 2012).

## One example: opportunistic reuse in the number line and the timeline

In blending as in neural reuse, a given item, once identified as potentially useful, is integrated into the network under construction. If necessary and possible, the item is adjusted for optimization in its new function. If it works, the item is kept in the network, although it still remains available for its older functions. Networks and their components are discarded and entrenched in a dynamic, extremely agile process. What is going on here is not direct transfer of structure, but rather the construction of a new whole with old pieces. The novel properties are not borrowed from the structures being reused, but result from their performance in a new network.

Among other examples, Anderson (2010) illustrates this with the spatial-numerical association of response codes (SNARC) effect, that is, a mental number line in which numerals are arrayed from left to right, in order of increasing magnitude (Dehaene et al., 1993). As Anderson explains, there is no metaphoric mapping or perceptual grounding here: in sensorimotor experience, magnitude may increase with height (the more is up metaphor), but not laterally, and certainly not in the direction of writing. Numerals do not inherit the structure of the spatial shifting mechanism: the left-to-right line has been picked opportunistically, and integrated with numeral magnitude, simply because the resulting blend meets the requirements of the task. We could add to Anderson's argument by pointing out that the mental number line, as a symbolic device, needed considerable cultural time to emerge: it was only invented in seventeenth-century Europe, although awareness of potential correspondances between numbers and spatial relations dates back to Babylon (Núñez, 2009). It took thousands of years for the pattern to find an appropriate niche, alongside a representational format that would ensure its transmission.

The number line is what gets called a *material anchor for a conceptual blend* (Hutchins, 2005). A perceptual structure is used as an input in the integration process. In the blend, perceptual relations are fused with conceptual relations. Now consider a very similar case. Varied psycholinguistic evidence shows that processing temporal expressions causes the automatic activation of a mental timeline, also running from left to right in cultures with that writing system (Torralbo et al., 2006; Weger and Pratt, 2008; Santiago et al., 2010). Blending theorists have revised the time is space metaphor, and shown that it is a complex network that produces a motion scene with special rules and constraints, designed to facilitate the representation of time: all observers are on the same spot, all objects move along the same path, and spatial relations can even be modified by the emotional attitude of the observer: “Monday is almost here, but Friday is so far away” (Fauconnier and Turner, 2008). The straight line is particularly useful for anchoring this blended scene. The result is a graphic representation with novel properties, which allow us to see diachrony at a glance, to divide it easily into periods, to represent events as dots, etc.

The timeline is a generic integration template that blends at least four inputs: time and time measures, spatial extent, objects, and events (Coulson and Cánovas, 2013). Spatial shifting from left to right is absent from all these components, but it happens to facilitate the task immensely, and thus it is imported to the blend, for local purposes. The pattern is not functional as a metaphor in language, where past and future are not on the left or right, but it is extremely productive in gesture (Casasanto and Jasmin, 2012), where the lateral axis is more easily available. Again, the timeline has a long history of failed cultural representations behind it (Rosenberg and Grafton, 2010), but, once the blending template found its niche, it is reused time and again, and can even be adapted for representing complex emotions and creating sophisticated poetic effects (Cánovas and Jensen, 2013). Metaphor is useful, but not enough to understand the timeline: a broader framework of reuse and integration is needed.

## What kind of model we need

BT researchers have indeed identified many recurrent patterns and theorized about them, but we still lack a general framework for generic integration templates (some work along those lines: Fauconnier and Turner, 2002; Fauconnier, 2009; Cánovas, 2010, 2011; Turner, 2014). A fruitful interaction with research on neural reuse can impose further constraints and requisites than those observed in the semantic or semiotic analyses. A model of conceptual mapping habits fully compatible with neural reuse may include the following:

- Network thinking (Mitchell, 2006) rather than direct binary transfer.
- Flexibility in the activation, selection, and integration of conceptual and neural patterns.
- Focus on emergence.
- Emphasis on competing optimality principles, e.g., a left-to-right straight line leaves aside many relevant aspects of time or magnitude, but its functionality is privileged.
- Detailed examination of how context and goals, including cultural diachrony, shape the process of integration.
- A model of entrenchment not based on ontological projection, but on the idea of “attaining a niche” through instance-based learning and context-sensitive usage.

### Conflict of interest statement

The authors declare that the research was conducted in the absence of any commercial or financial relationships that could be construed as a potential conflict of interest.
